# Complementary and Integrative Oncology in the Cross-Cultural Region of the Middle East and South Asia

**DOI:** 10.1155/2012/940961

**Published:** 2012-04-17

**Authors:** Eran Ben-Arye, Barrie Cassileth, Peter Heusser, Fatma Afifi, Bashar Saad, Senthamil R. Selvan

**Affiliations:** ^1^Integrative Oncology Program, The Oncology Service and Lin Medical Center, Clalit Health Services, Haifa 35152, Israel; ^2^Complementary and Traditional Medicine Unit, Department of Family Medicine, Technion-Israel Institute of Technology, Haifa 32000, Israel; ^3^Integrative Medicine Service, Memorial Sloan-Kettering Cancer Center, New York, NY 10021, USA; ^4^Department of Medicine, Center for Integrative Medicine, Faculty of Health, University of Witten/Herdecke, 58313 Herdecke, Germany; ^5^Department of Pharmaceutical Sciences, Faculty of Pharmacy, University of Jordan, Amman 11942, Jordan; ^6^Qasemi Research Center, Al-Qasemi Academic College, Baga Algharbiya 30100, Israel; ^7^Department of Biology and Biotechnology, Arab American University, Jenin, Palestine; ^8^Department of Medical Oncology, Thomas Jefferson University, 1015 Walnut Street, Suite 1008, College Bldg Philadelphia, PA 19107, USA

 The integration of traditional, complementary, and integrative medicine (CIM) in contemporary cancer care is an emergent field of clinical practice and research throughout the world. The use of herbs, nutrition, mind-body, and spiritual practices is deeply rooted in the cross-cultural mosaic of Middle Eastern and South Asian nations. The concept of integrative oncology has emerged in the last decade to signify the need to amalgamate traditional and complementary medicine practices with evidence-based research aiming to improve supportive cancer care. The integration of ancient *roots* with contemporary scientific *sprouts* is not merely a metaphor but a significant tool for promoting holistic patient-centered care that emphasizes patients' well-being rather than focusing merely on cancer cells and disease-centered terminology. Indeed, the remarkable achievements in contemporary integrative oncology only emphasize the need for a patient-tailored strategy of care attuned to the individual's biophysical, psychological, social, cultural, and spiritual needs and concerns. The integrative challenge is how to provide an evidence-based consultation and supportive treatment to patients who confront fear at the moment of breaking the bad news of cancer diagnosis; how to improve their well-being during chemotherapy, radiation, surgical, or palliative treatment; and how to support patients and their care providers along the survivorship pathway or across the threshold of life. In daily practice, integrative oncology may be employed to reduce nausea and vomiting (e.g., use of the traditional Ayurvedic and Chinese herb *Zingiber officinale* known as ginger [1]), to alleviate pain (e.g., acupuncture [2]), and to improve fatigue (e.g., exercise, relaxation and body awareness training combined with massage [3]), mood disturbances (manual modalities [4]), and many other disease symptoms and chemotherapy side effects. Integrative oncology is also challenged by the need to obtain CIM safety (e.g., awareness of the risks of herbal-chemotherapy interactions) and high-quality standards of CIM supplements as well as professional training of integrative practitioners. Last but not least, these fundamental elements need to be enhanced by open communication channels between CIM practitioners, oncologists, and other health care providers in order to conclude a comprehensive integrative approach based on vibrant multidisciplinary discourse. Hence, the concept of *integrative oncology* goes far beyond *traditional*, *alternative*, or *complementary* practice, signifying a call for holistic practice, a whole that is larger than the sum of its scientific, clinical, and humanistic parts.

The paper by H. Zaid et al. featured in this special issue reviews the concept of traditional Islamic medicine with regard to herbs with potential anticancer activity. This paper illuminates the importance of bridging ancient knowledge rooted in Greco-Arabic medicine and contemporary research. But, in addition to the extensive review presented by H. Zaid et al., this paper is also distinctive thanks to the contribution of the two other authors in this collaborative Israeli-Palestinian paper. Notable is the contribution of M. Silbermann, the director of the US National Cancer Institute-affiliated Middle-East Cancer Consortium (MECC), who has succeeded over the last 15 years in promoting supportive care collaborations that have included joint integrative oncology projects between MECC (Egypt, Israel, Palestinian Authority, Jordan, Turkey, and Cyprus) and other Middle-Eastern countries [5, 6]. 

 The paper by E. Ben-Arye presents an integrative oncology program operated within conventional oncology services in northern Israel aimed at improving patients' quality of life during chemotherapy and advanced cancer. The authors address barriers to integration of traditional and complementary medicine in supportive care of Arab patients and propose six practical recommendations aimed at improving patients' access to integrative supportive care as well as compliance with treatments. This paper emphasizes the need to base integrative oncology on a sensitive cross-cultural approach that takes into consideration social, cultural, and spiritual elements.

The paper by M. Schaltz et al. intensifies this cross-cultural theme by reflecting on palliative care from the perspective of Jewish and Islamic traditions. The collaboration in this paper between M. Schultz and K. Baddarni, two scholars in spiritual supportive care in northern Israel, highlights the richness of therapeutic dialogue between Muslim and Jewish health care providers who share faith in the role of the integrative dialogue. This paper summarizes ethical, religious, and spiritual insights gleaned in the management of patients in the community-centered Al-Taj organization and in the oncology department in Rambam health care campus, which is named for the renowned Jewish physician Maimonides.

The paper authored by I. Cantarero-Villanueva et al. from Granada presents the flavor of the ancient cities of southern Spain, the backdrop for the Golden Age of collaborative Muslim and Jewish physicians, including Maimonides and the followers of Ibn-Sina, the most prominent Islamic medicine scholar. In this paper, the authors evaluated, in a randomized controlled trial, the effects of a multimodal exercise and massage program on the well-being of breast cancer survivors. The reduced fatigue, tension, depression, and improved vigor and muscle strength after intervention and 6 months after discharge are remarkable and support the need for other rigorous trials in the integrative oncology field.

 Moving from West to East across the Mediterranean and West Asia, the paper by P. Puataweeponge et al. presents the notion of complementary medicine in Bangkok, Thailand. The authors present a study regarding CAM use by a large cohort of cancer patients attending outpatient radiotherapy treatment in Thailand. The high prevalence of CAM use of more than 60% is notable in light of the patient-oncologist communication gap illustrated by the high prevalence (58.3%) of patients who did not disclose CAM use to their doctors. This communication aspect should raise concern when 9.4% of patients in this study reported side effects of CAM treatments. Moreover, this study emphasizes the need for a paradigm shift from CAM (with emphasis on *alternative*) to CIM (with emphasis on *integrative*) that will enable patients and physicians to discuss complementary use in an open nonjudgmental context.

 Two papers in this issue present the fundamental *in vitro* research elements needed to base any clinical integrative oncology activity. Zhang et al. from China report. the anticancer effects of the photochemical Zerumbone, isolated from the plant *Zingiber zerumbet* Smith. Although additional rigorous studies are warranted, the promising apoptosis induction effect of this plant on pancreatic carcinoma cell lines may suggest that ginger and related plants may have anticancer properties in addition to their beneficial effect in chemotherapy-related nausea and vomiting. In the paper by Y. H. Liu et al. from neighboring Taiwan, they studied Abrin, a protein purified from the seeds of *Abrus precatorius*, and reported that prohibitin, a tumor-suppressing protein, plays a role in abrin-induced apoptosis. These findings join the growing number of promising studies in integrative oncology that may support development of new and, in some cases, traditional medicine-based, therapeutic agents and modalities for the benefit of patients with cancer across the globe.

## References

[1] Pillai AK, Sharma KK, Gupta YK, Bakhshi S (2011). Anti-emetic effect of ginger powder versus placebo as an add-on therapy in children and young adults receiving high emetogenic chemotherapy. *Pediatric Blood and Cancer*.

[2] Pfister DG, Cassileth BR, Deng GE (2010). Acupuncture for pain and dysfunction after neck dissection: results of a randomized controlled trial. *Journal of Clinical Oncology*.

[3] Adamsen L, Quist M, Andersen C (2009). Effect of a multimodal high intensity exercise intervention in cancer patients undergoing chemotherapy: randomised controlled trial. *BMJ*.

[4] Listing M, Reißhauer A, Krohn M (2009). Massage therapy reduces physical discomfort and improves mood disturbances in women with breast cancer. *Psycho-Oncology*.

[5] Ben-Arye E, Schiff E, Hassan E (2012). Integrative oncology in the Middle East: from traditional herbal knowledge to contemporary cancer care. *Annals of Oncology*.

[6] Ben-Arye E, Ali-Shtayeh MS, Nejmi M (2012). Integrative oncology research in the Middle East: weaving traditional and complementary medicine in supportive care. *Supportive Care in Cancer*.

